# Evolved-Cooperative Correntropy-Based Extreme Learning Machine for Robust Prediction

**DOI:** 10.3390/e21090912

**Published:** 2019-09-19

**Authors:** Wenjuan Mei, Zhen Liu, Yuanzhang Su, Li Du, Jianguo Huang

**Affiliations:** 1 Department of Instrument Science and Technology, University of Electronic Science and Technology of China, Chengdu 611731, China; meiwenjuan@std.uestc.edu.cn (W.M.); scdliu@uestc.edu.cn (Z.L.); summer_christ@163.com (L.D.); xlhjg@uestc.edu.cn (J.H.); 2 Department of Applied Linguistics, University of Electronic Science and Technology of China, Chengdu 611731, China

**Keywords:** correntropy, information theory extreme learning machine, evolved cooperation

## Abstract

In recent years, the correntropy instead of the mean squared error has been widely taken as a powerful tool for enhancing the robustness against noise and outliers by forming the local similarity measurements. However, most correntropy-based models either have too simple descriptions of the correntropy or require too many parameters to adjust in advance, which is likely to cause poor performance since the correntropy fails to reflect the probability distributions of the signals. Therefore, in this paper, a novel correntropy-based extreme learning machine (ELM) called ECC-ELM has been proposed to provide a more robust training strategy based on the newly developed multi-kernel correntropy with the parameters that are generated using cooperative evolution. To achieve an accurate description of the correntropy, the method adopts a cooperative evolution which optimizes the bandwidths by switching delayed particle swarm optimization (SDPSO) and generates the corresponding influence coefficients that minimizes the minimum integrated error (MIE) to adaptively provide the best solution. The simulated experiments and real-world applications show that cooperative evolution can achieve the optimal solution which provides an accurate description on the probability distribution of the current error in the model. Therefore, the multi-kernel correntropy that is built with the optimal solution results in more robustness against the noise and outliers when training the model, which increases the accuracy of the predictions compared with other methods.

## 1. Introduction

With the rapid development of powerful computing environments and rich data sources, artificial intelligence (AI) technology such as neural networks [1,2,3], adaptive filtering [4,5,6] and evolutionary algorithms [7,8,9] has become increasingly more applicable for forecasting problems in various scenarios, such as medicine [10,11,12], economy [13,14,15] and electronic engineering [16,17,18]. The methods have acquired high reputations due to their great approximation abilities.

Although AI methods perform well when solving real world problems, most corresponding models adapt the mean squared error (MSE) as the criterion for training hidden nodes or building the cost functions, assuming that the data satisfy a Gaussian distribution. Moreover, the MSE is a global similarity measure where all the samples in the joint space have the same contribution [19]. Therefore, the MSE is likely to be badly affected by the noise and outliers that are hiding in the samples and this happens commonly in applications, such as speech signals, images, real-time traffic signals and electronic signals from ill-conditioned devices [20,21,22]. Therefore, MSE-based models are likely to result in poor performance in real world applications.

To conquer the weaknesses of the least mean squares (LMS), over the past decades, a number of studies have proposed methods to improve the robustness of the model against the noise and outliers that are contained in the data [23,24,25,26,27]. Among the existing technologies, M-estimators have been the focus of many academic studies. By detecting the potential outliers during training procedures, the M-estimator can eliminate the negative influences from the output weights that adversely affect the predictions [28]. Using these advantages, Zhou et al. [29] proposed a novel data-driven standard least-squares support vector regression (LSSVR) applying the M-estimator, which reduces the interference of outliers and enhances the robustness. However, there are difficulties accessing clean learning data without noises so that the application on the M-estimator-based forecasting models based is limited.

Recently, information theoretic learning (ITL) has drawn considerable attention due to its good performance avoiding the effect of the noise and outliers [30,31,32,33,34,35] and it has become an effective alternative to the MSE criterion. In [36], the authors presented a novel training criterion based on the minimum error entropy (MEE) to replace the MSE. By taking advantages of the higher order description on entropy, MEE has become superior for non-Gaussian signal processing compared with traditional training criteria. Inspired by the entropy and Parzen kernel estimator, Liu et al. [37] proposed an extended definition of the correlation function for random processes using a generalized correlation function, known as correntropy. Although different from global measurements, such as the mean squared error (MSE), the correntropy is regarded as a local similarity measurement where its value is primarily determined by the kernel function along x = y line [38], leading to high robustness against noise and outlies. Moreover, the correntropy has many great properties such as symmetry, nonnegativity and boundness. Most of all, it is easy to form convex cost functions based on the correntropy, which is very convenient for training the models [39,40,41,42]. Therefore, the correntropy has been widely used in forming robust models [43,44,45].

To enhance the forecasting ability of the model, in [46], the correntropy was introduced into the affine projection (AP) algorithm to overcome the degradation of the identification performance with impulsive noise environments. From the simulation results, it is easy to verify that the proposed algorithm has achieved better performance than other methods. Another approach to improve the robustness via the correntropy is enhancing the feature selection efficiencies [47,48,49]. In [50], the kernel modal regression and gradient-based variable identification were integrated together using the maximum correntropy criterion, which guarantees the robustness of the algorithm. Additionally, in [51], a novel principal component analysis (PCA), based on the correntropy and known as the correntropy-optimized temporal PCA (CTPCA), was adapted to enhance the robustness for rejecting the outlier. The outlier improves the models training in simulation experiments. In addition to providing the extractions of the features in neural networks and filtering methods, the correntropy turns out to be a powerful tool for developing robust training methods that generate and adjust the weights in the model. In [52], Wang et al. introduced a feedback mechanism using the kernel recursive maximum correntropy to provide a novel kernel adaptive filters known as the kernel recursive maximum correntropy with multiple feedback (KRMC-MF). The experiments show that the generated filters have high robustness against outliers. In [53], Ahmad et al. proposed the correntropy based conjugate gradient backpropagation (CCG-BP), which can achieve high robustness in environments with both impulsive noise and heavy-tailed noise distributions. Unfortunately, most of the neural networks have to adjust the weights of each node during each training iterations which wastes time during the training process.

Recently, forecasting models with parameters that are free from adjustments have gained increasingly more attention due to their fast training speeds for the models [54,55,56]. Combined with the correntropy, these algorithms have shown great potential in real-world applications. For example, Guo et al. [57] developed a novel training method for echo state networks (ESNs) based on a correntropy induced loss function (CLF), which provides robust predictions for time-series signals. Similar to ESNs, extreme learning machines (ELMs) have received great attention on fast learning due to the random assignments of the hidden layer and being equipped with simpler structures, such as single layer feedback networks (SLFNs) [58,59,60]. It has been proven that the hidden nodes can be assigned with any continuous probability distribution, while the model satisfies the universal approximation and classification capacity [61]. In particular, the extreme learning machine has been applied and received a high reputation for predicting production processes [62,63], system anomalies [64], etc. [65]. In [66], the authors first developed the correntropy-based ELM that uses the regularized correntropy criterion in place of the MSE with half quadratic (HQ) optimization which is called the regularized correntropy criterion for an extreme learning machine (RCC-ELM). Later, Chen et al. [67] extended the dimensions of the correntropy by combining two kinds of correntropy together to enhance the flexibility of the model to generate more robust ELM called ELM by maximum mixture correntropy criterion (MMCC-ELM). The experimental results show that the learning method performs better than the conventional maximum correntropy method. Although the RCC-ELM and MMCC-ELM possess high robustness compared with other ELM methods, the corresponding correntropy is constrained by no more than two kernels. The kernel bandwidth required for the assignments by users in advance is likely to degrade the model due to the improper description on the probability distribution of the signal with the correntropy.

To conquer the weakness of the existing correntropy-based ELMs, this paper focuses on providing a more robust predicting model with adaptive generation based on multi-kernel correntropy which can bring an accurate description of the current errors of ELM. This study developed a more flexible and robust forecasting ELM based on a newly developed adaptive multi-dimension correntropy using evolving cooperation. In the proposed method, the output weights of the ELM are trained based on the maximum multi-dimension correntropy with no constraints on the dimensions of the kernels. To achieve the most appropriate assignment of the parameters of each kernel in the correntropy, a novel evolving cooperation method is developed to concurrently optimize the bandwidths and the corresponding influence coefficients to achieve the best estimations of the residual errors of the model. Furthermore, the training approach has been developed based on the properties of the multi-dimension correntropy. The main contribution of the paper can be summarized as follows.

- The proposed method develops a novel correntropy criterion with multiple kernels to improve the flexibility for depicting the probability distribution of the current error of the predicting model. Then, a convex cost function has been developed based on the multiple kernel correntropy, which can provide a more robust training strategy for ELMs, resulting in high performance on the predictions against noise and outliers.
- To accurately describe the probability distribution of the current error, the proposed method develops a cooperating evolution strategy to adaptively generate proper bandwidths and coefficients to suit the error distribution which enhances the accuracy on the approximation for the correntropy, leading to more robust training.

The experiments compare the performance of the proposed method and several state-of-art methods using both simulated data and real-world data, which show that the proposed method obtains more the robust predictions than other methods. Finally, the proposed method is incorporated into the forecasting model for the current transfer ratio (CTR) signals for the optical couplers, and it achieves high accuracies and robustness.

The rest of the paper is as follows. The next section introduces the framework of the proposed method and multi-dimension correntropy. Section 3 describes the evolved cooperation for the kernels with multi-dimension correntropy and Section 4 provides the training procedures of the forecasting model. Then, Section 5 estimates the performance of the proposed method using both simulation data and real-world applications. Finally, the conclusion is drawn in Section 6.

## 2. The Framework of the Proposed Method

**Figure 1:**
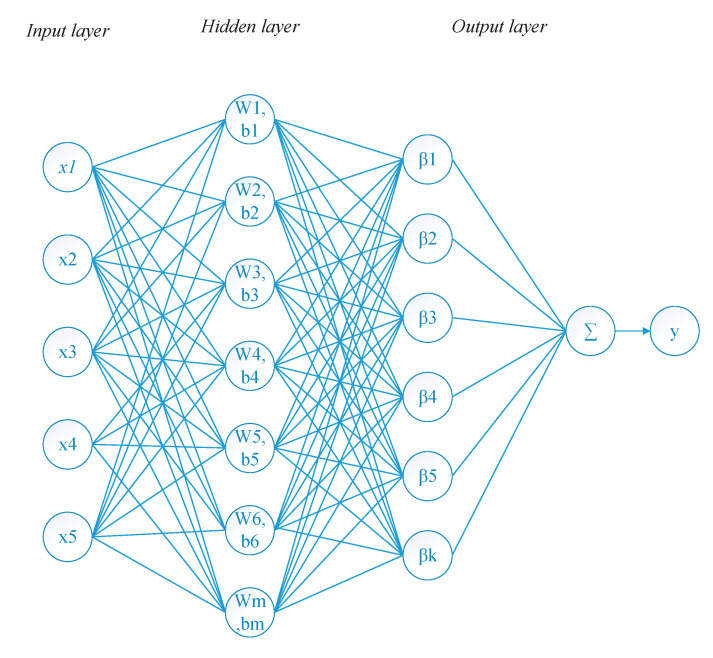
The structure of the prediction model.

The structure of the prediction model that is built using the proposed method is similar to those of other ELM-based methods. Figure 1 shows the basic structure of the method. Generally, the network includes one input layer, one hidden layer and one output layer. The hidden output is calculated using the given input vectors and the weights and the biases of the hidden nodes which are randomly assigned [54]:

(1) h=f(wx+b)

where *f*(.) is the activation function and (***w***,***b***) are the weights and bias of the hidden nodes.

With the hidden layer, the network can simulate any kind of function by generating the output weights with the least mean squares (LMS) The cost function is calculated as follows [58]:

(2) $$J_{\mathrm{LS}}=||Y-T||$$

where **T** is the expected output and **Y** is the predicted output of the model. **Y** calculated with the hidden outputs h and the output weights ***β*** as follows:

(3) Y=βh

Therefore, the output layer is calculated as follows:

(4) $${\beta =(H}^{T}{H)}^{-1}H^{T}T$$

Further, to constrain the output weights, the output layer is calculated as follows:

(5) $${\beta =(H}^{T}{H+\lambda I)}^{-1}H^{T}T$$

where λ is the constraining coefficient.

Although the output weights that are calculated by Equation (4) or Equation (5) can provide good predictions using the training data, the model has suffered with the outliers and noises in the data which negatively affect the predictions. To overcome the problem, the correntropy, as a high order similarity measurement, has been used in some recently developed methods.

In [62], the cost function built using the correntropy as follows:

(6) $$J_{\mathrm{RCC}}{=\max}_{\beta }[\overset{N}{\underset{p=1}{\sum }}G\left(t_{p}-h\beta \right)-\lambda ||\beta ||]$$

where G(**t_p_** − ***hβ***) is the Gussian kernel calculated as follows:

(7) $$G\left(t_{p}-h\beta \right)=\exp(-\frac{{(t}_{p}-h\beta )}{{2\sigma }^{2}})$$

where σ is the bandwidth of the kernel.

Therefore, the output layer is calculated as follows:

(8) $${\beta =(H}^{T}{\Lambda H-\lambda I)}^{-1}H^{T}\Lambda T$$

where ***Λ*** is the diagonal matrix of the local optimal solution. It is calculated as follows:

(9) $$\alpha_{p}^{\tau +1}{=-G(t}_{p}-h\beta )$$

To further improve the flexibility of the correntropy, the cost function with a mixed correntropy is defined in [67] as follows:

(10) $$J_{\mathrm{MMCC}}=1-\frac{1}{N}\overset{N}{\underset{i=1}{\sum }}\left[{\alpha G}_{\sigma_{1}}\left(e_{i}\right)+\left(1-\alpha \right)G_{\sigma_{2}}\left(e_{i}\right)\right]+\lambda ||\beta ||$$

Therefore, the output is calculated as follows:

(11) $${\beta =(H}^{T}{\Lambda H+\lambda^{\prime }I)}^{-1}H^{T}\Lambda T$$

where the $\lambda^{\prime }=2N\lambda$ and ***Λ*** is the diagonal matrix with elements calculated as follows:

(12) $$\Lambda_{ii}={\alpha /\sigma }_{1}G_{\sigma_{1}}\left(e_{i}\right)+\left(1-\alpha \right){/\sigma }_{2}G_{\sigma_{2}}\left(e_{i}\right)$$

**Figure 2:**
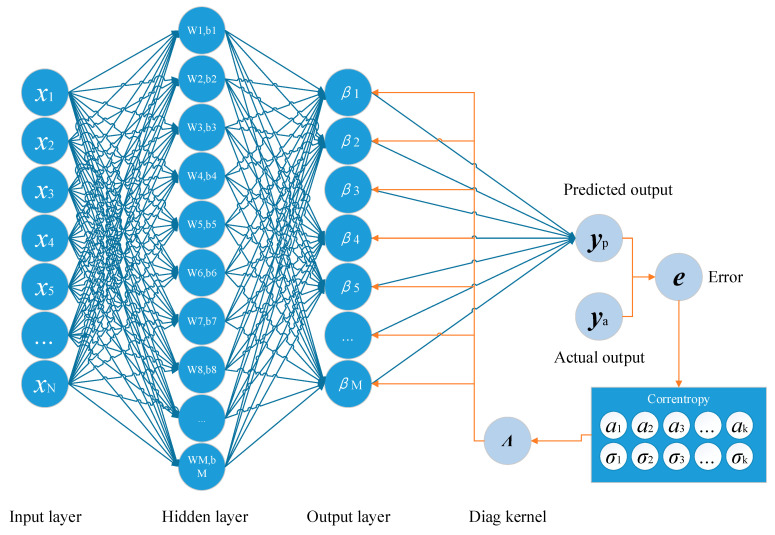
The framework of the proposed method.

With two coefficients, Equation (9) gives a more accurate estimation of the costs of the output layer, leading to a higher robustness of the model. Although Equations (7) and (9) can acquire better local similarity measurements compared with Equation (5), both criterions limit the correntropy into two kernels, leading to an inappropriate description on the probability distribution of the data. Additionally, the bandwidths and the coefficients must be assigned by users, thus limiting the performance of the corresponding model in real world applications which can be badly affected since the bandwidths are not suitable for the estimation of the correntropy. To provide a more flexible criterion for the training strategy with a more appropriate description of the probability distribution of the data, the proposed method develops a multi-kernel correntropy criterion that is calculated as follows:

(13) $$k\left(T-\beta H\right)=\overset{K}{\underset{i=1}{\sum }}\alpha_{i}G_{\sigma_{i}}\left(T-\beta H\right)$$

where α_i_ is the influence coefficients controlling the weight of each kernel. By using multiple kernels to construct the correntropy, the proposed method brings a more accurate approximation on the probability distribution of the samples, leading to a high prediction performance of the model. Based on the corretropy using Equation (13), the proposed method built a convex cost function for training the output weights, which has been analyzed in Section 4. For the suitable assignments of the parameters in Equation (13), a novel generation strategy using an evolved cooperating process based on SDPSO with the MIE to generate the parameters adaptively has been developed. Therefore, the framework of the proposed method can be summarized in Figure 2. The proposed method developed an evolved-cooperation strategy to generate the optimized solution of the influence coefficients and the bandwidths which suits the distribution of the prediction errors. To achieve an accurate estimation, the bandwidth was generated based on switching delayed particle swarm optimization (SDPSO) [68] and the influence coefficients were calculated based on the cost function for estimating the probability distribution function of errors.

The basic procedures of the method are as follows. Supposing that the input vector of the samples is represented as ***x*** = {x_1_, x_2_, …, x_N_}, calculate the output of hidden nodes with randomly assigned weights and biases as Equation (1). Then, adapt the cooperating evolution technology for training the output weights. For each iterations of the evolution, the output of the predicting model can be generated using Equation (3). Compared with the actual outputs, the predicted outputs result in current error ***e*** with the model. Based on the current error ***e***, the proposed method makes the best assignments of the bandwidths in the correntropy with SDPSO and accesses the optimal coefficients based on MIE. This is shown in the next section. Using the generated correntropy, a list of diagnostic kernels can be calculated which effects the updating of the output layer to reach higher accuracy. This is presented in Section 4. The processes stop when the cost function of the model is stable.

More details are presented in the next section.

## 3. The Cooperating Evolution Process for the Bandwidth and Influence Coefficients of the Kernel

For the correntropy that is defined by Equation (12), the bandwidth and the influence coefficients are for the similarity measurements since the bandwidths act as the zoom lens for the measurements and the coefficients determine the effect that each kernel has on the estimation of the correntropy according to the assigned bandwidth. They are defined as follows:

(14) $${\sigma =\{\sigma }_{1}{,\sigma }_{2}{,\sigma }_{3}{,\ldots ,\sigma }_{M}\}$$

(15) $$A=\left\{\alpha_{1},\alpha_{2},\alpha_{3},\ldots ,\alpha_{M}\right\}$$

Therefore, the bandwidth and the influence coefficients should be carefully assigned to match the probability distribution of the samples to achieve the best effect of the correntropy on generating the output weights of the prediction model. Since the correntropy depicts the probability distribution of the distance between the actual output and the model response, the bandwidth and the coefficients are able to form the probability distribution (pdf) function as follows:

(16) $$\hat{f}(e)=\sum_{i=1}^{N}\alpha_{1}G_{\sigma_{1}}(e_{i})+\alpha_{2}G_{\sigma_{2}}(e_{i})+\cdots +\alpha_{N}G_{\sigma_{n}}(e_{i})$$

In applications, the real joint probability distribution for the cases are unknown. Therefore, the joint pdf can only be estimated for a finite number of samples{(ti,yi)}, where i = 1, 2, …, N:

(17) $$f\left(e\right)=\frac{1}{N}{g(\{(t}_{k}{,y}_{k})|\left|t_{k}-y_{k}\right|=e\})$$

where g(**S**) is the cardinal number of the set **S**.

Using the kernel contrasts between the pdf estimated with the assigned parameters and the pdf estimated using the data, the least mean integrated error (MIE) can be calculated as follows:

(18) $$\mathrm{MIE}=E(\int \left(\hat{f}(e)-f(e\right){)}^{2}de)$$

Based on the MIE, the performance of the bandwidth and coefficients can be estimated using the contrasts with the pdf from the data. Therefore, the optimization of these parameters can be transformed to finding the solution with the minimum MIE.

In the proposed method, the switching delay particle swarm optimization is adapted to search for the best bandwidth. To achieve this, the particles are initialized with a list of potential bandwidth setting σc = {σ_c,1_, σ_c,2_, …, σ_c,N_}. With respect to each bandwidth of the particle, the velocities for the evolution of the particles are defined as follows:

***vσ***_c_ = {vσ_c,1_, vσ_c,2_,…, vσ_c,N_} (19)

Meanwhile, the influence coefficient is denoted as vector ***A***:

(20) $$A_{c}=\left\{\alpha_{c,1},\alpha_{c,2},\alpha_{c,3},\ldots ,\alpha_{c,M}\right\}$$

where *α*_i_ is the influence coefficient according to σ_c,i._

Since the samples provide disperse values of the outputs, the pdf from the data is estimated using the discrete version of Equation (16):

(21) $$F=\{f(m_{1}),f(m_{2}),\ldots ,f(m_{k})\}$$

(22) $$f\left(m\right)=\frac{1}{N}{g(\{(t}_{k}{,y}_{k})|m-\varepsilon \leq \left|t_{k}{-y}_{k}\right|\leq m+\varepsilon \})$$

where the vector **m** = {m_1_, m_2_, …, m_k_} is a list of values that satisfy m_1_ < m_2_ < … < m_k_ and |m_i_ − m_i−1_| = ε. ε is the step length of the estimation.

Accordingly, the values from Equation (15) with respect to **m** are equivalent to the following set:

(23) $$\hat{F}=\{\hat{f}(m_{1}),\hat{f}(m_{2}),\cdots ,\hat{f}(m_{k})\}$$

They can be calculated as:

(24) $$\hat{F}=AK$$

where ***K*** is the kernel matrix, which is as follows:

(25) $$K=\left[\begin{array}{cc} \begin{array}{cc} G_{\sigma_{1}}{(e}_{1}) & G_{\sigma_{1}}{(e}_{2})\\ G_{\sigma_{2}}{(e}_{1}) & G_{\sigma_{2}}{(e}_{2}) \end{array} & \begin{array}{cc} \ldots & G_{\sigma_{1}}{(e}_{N})\\ \ldots & G_{\sigma_{2}}{(e}_{N}) \end{array}\\ \begin{array}{cc} \vdots & \vdots \\ G_{\sigma_{M}}{(e}_{1}) & G_{\sigma_{M}}{(e}_{2}) \end{array} & \begin{array}{cc} \ddots & \vdots \\ \ldots & G_{\sigma_{M}}{(e}_{N}) \end{array} \end{array}\right]$$

By inserting Equations (20) and (22) into Equation (17), the following cost function can be obtained:

(26) $${\mathrm{MIE}=(\mathrm{AK}-F)(\mathrm{AK}-F)}^{T}$$

Then, the following differential equations with respect to **A** are calculated:

(27) 2(AK−F)=0

Therefore, the coefficient can be calculated using the assigned bandwidth as follows:

(28) $$A=FK^{T}{\left(KK^{T}\right)}^{-1}$$

Since each particle contains one solution for the kernels’ parameters, the personal best solution pσ and the global best solution gσ is updated by minimizing the costs. Then, the particles are updated as follows:

(29) $${v\sigma }_{c}\left(k+1\right){=\mathrm{wv}\sigma }_{c}{+c}_{1}\left(k\right){\times r}_{1}\left(p\sigma \left(k-\left(k\right){)-\sigma }_{c}\left(k\right)\right)\right){+c}_{2}\left(k\right){\times r}_{2}\left(g\sigma \left({k-\tau }_{2}\left(k\right)-\sigma_{c}\left(k\right)\right)\right)$$

(30) $$\sigma_{c}\left(k+1\right){=\sigma }_{c}\left(k\right){+v\sigma }_{c}\left(k+1\right)$$

where c_1_(k) and c_2_(k) are the acceleration coefficients and τ_1_(k) and τ_2_(k) are the time delays. All the parameters are adjusted based on the evolution factor, Ef, which determines the evolutionary states, and it is calculated as follows:

(31) $${\mathrm{Ef}=(d}_{g}{-d}_{\min}{)/(d}_{\max}{-d}_{\min})$$

where d_g_ is the global best particle among the mean distance. It is calculated as:

(32) $$d_{g}=\frac{1}{N}\overset{N}{\underset{i=1}{\sum }}{||\sigma }_{c,i}-g\sigma ||$$

With the estimate on Ef, the parameters can be selected as shown in Table 1.

The final solution of the bandwidth and the influence coefficients are determined as the solution that minimizes the costs during the evolution procedures.

In summary, the cooperative evolution process is shown in Algorithm 1. First, the bandwidth and the corresponding velocity of each particle are randomly assigned. Then, for each iteration of the process, the influence coefficients are evolved using the bandwidth based on the MIE and the particles are updated using the cost function. Finally, the algorithm finds the best solutions for the bandwidth and the influence coefficients, from which the kernel depicts the pdf from the data. Based on the generated kernel, the correntropy can lead to a model with good robustness.

**Algorithm 1** Evolved cooperation for the kernel parameters**Input:** the samples {x_i_,t_i_},i = 1, 2, …, N **Output:** the vector of bandwidth σ and the vector of influence coefficients ***A***  **Parameters:** the step length and the number of iterations L  **Initialization:** Set the cost function of the best solution MIE_best_ to ∞ and randomly assign the bandwidth of the kernels **σ**_c_ = {σ_c,1_, σ_c,2_, …, σ_c,N_} and the corresponding velocity **vσ**_c_ = {vσ_c,1_, vσ_c,2_, …, vσ_c,N_}. 1: **for** k = 1, 2, … L **do** 2:    Generate the best influence coefficients ***A***c using Equation (26) for each particles. 3:    Calculate value of cost function for each particle MIEc based on Equation (24) 4:    Update the personal best solution pσ and the global best solution gσ based on minimizing the cost function. 5:    Calculate the Ef of the iteration with Equation (29) 6:    Access the parameters for evolution based on Table 1 7:    Update the swarm with Equations (27) and (28) 8: **end for** 9: Return the global best bandwidth gσ and the corresponding influence coefficients

## 4. Training the Extreme Learning Machine Using the Multi-Dimension Correntropy

To improve the robustness of the extreme learning machine, in the proposed method, the training procedure of the output layer as Equation (5), is replaced by the developed calculation using the mixture correntropy that is generated using the evolved kernel from Section 3. The loss function for the output layer is developed according to the following properties.

**Property** **1.** *K(**Y**,**T**) is symmetric, which means the following: K(**Y**,**T**) = K(**T**,**Y**).*

**Property** **2.** *K(**T**,**Y**) is positive and bounded, which means the following: 0 < K(**Y**,**T**) < = 1 and K(**T**,**Y**) = 1 if and only if **T** = **Y**.*

**Property** **3.** *K(**T**,**Y**) involves all the even moments of e, which means the following:*

(33) $$K\left(T,Y\right){=E[e}^{2n}]\overset{\infty }{\underset{n=0}{\sum }}\frac{{\left(-1\right)}^{n}\sum_{i=1}^{M}\alpha_{i}\sigma_{i}^{2n}}{2^{n}\prod_{i=1}^{M}{{(\sigma }_{i})}^{2n}n!}$$

**Property** **4.** *When the first bandwidth is large enough, it satisfies the following:*

(34) $$K\left(T,Y\right)\approx \overset{M}{\underset{i=1}{\sum }}\alpha_{i}-\frac{\sum_{i=1}^{M}\alpha_{i}\sigma_{i}^{2}}{2\prod_{i=1}^{M}\sigma_{i}^{2}}{E[e}^{2}]$$

**Proof.**  For $\underset{x\to 0}{\lim}\exp\left(x\right)\approx 1+x$, suppose that σ_1_ is large enough, K(**T**,**Y**) can be approximated as follows:

(35) $$K\left(T,Y\right){=\alpha }_{1}G_{\sigma_{1}}\left(e\right){+\alpha }_{2}G_{\sigma_{2}}\left(e\right){+\ldots +\alpha }_{m}G_{\sigma_{m}}\left(e\right)\;{=\alpha }_{1}\left(1-\frac{e^{2}}{{2\sigma }_{1}^{2}}\right){+\alpha }_{2}\left(1-\frac{e^{2}}{{2\sigma }_{2}^{2}}\right){+\ldots +\alpha }_{m}\left(1-\frac{e^{2}}{{2\sigma }_{m}^{2}}\right)\;=\overset{m}{\underset{i=1}{\sum }}\alpha_{i}-\frac{\sum_{i=1}^{M}\alpha_{i}\sigma_{i}^{2}}{2\prod_{i=1}^{M}\sigma_{i}^{2}}{E[e}^{2}]$$

that completes the proof. □

**Remark** **1.** *Based on Property 4, the mixed C-loss is defined as L(**T**,**Y**) = 1 − K(**T**,**Y**), which is approximately equivalent to the mean square error (MSE) with a large enough bandwidth.*

**Property** **5.** *The empirical mixed C-loss L(e) that is a function of e is convex at any point satisfying ${\left|\left|e\right|\right|}_{\infty }=\max\left|e_{i}\right|\leq \sigma_{1}$.*

**Proof.**  Build the Hessian matrix of the C-loss function L(e) with respect to e as follows:

(36) $$H_{L\left(e\right)=}\left[\frac{\partial L(e)}{\partial e_{i}\partial e_{j}}\right]{=\mathrm{diag}(\xi }_{1}{,\xi }_{2}{,\ldots ,\xi }_{N})$$

The elements of matrix ξ is calculated as follows:

(37) $$\xi_{i}=\overset{m}{\underset{i=1}{\sum }}\alpha_{i}\frac{\sigma_{i}^{4}-e_{i}^{4}}{N\sigma_{i}^{4}}G_{\sigma_{i}}\left(e_{i}\right)$$

It is obvious that $\xi_{i}$ is positive. Therefore, L(e) is convex. □

**Remark** **2.** *Using Property 4 and Property 5, the loss function of the output weights is based on the empirical mixed C-loss L(e) from the data observations, which can be defined as follows:*

(38) $$J=L\left(T,Y\right){+\lambda ||\beta ||}^{2}=1-\frac{1}{N}\overset{N}{\underset{i=1}{\sum }}\overset{M}{\underset{j=1}{\sum }}\alpha_{j}G_{\sigma_{j}}\left(e_{i}\right){+\lambda ||\beta ||}^{2}$$

*Based on Equation (38), the training criterion is generated for improvement on the robustness of the model.*

Taking the differential of the loss function, it is easy to get the following:

(39) $$\begin{array}{l} \frac{\partial J(\beta )}{\partial \beta }=0\\ -\sum_{i=1}^{N}\{[\sum_{j=1}^{M}\frac{\alpha_{j}}{\sigma_{j}^{2}}G_{\sigma_{j}}(e_{i})]e_{i}h_{i}^{T}\}+2N\lambda \beta =0\\ \sum_{i=1}^{N}\left(\varphi (e_{i})h_{i}^{T}h_{i}\beta -\varphi (e_{i})t_{i}h_{i}^{T}\right)+\lambda^{\prime }\beta =0\\ \sum_{i=1}^{N}(\varphi (e_{i})h_{i}^{T}h_{i}\beta +\lambda^{\prime }\beta =\sum_{i=1}^{N}\left(\varphi (e_{i})t_{i}h_{i}^{T}\right)\\ \beta ={\left[H^{T}\Lambda H+\lambda^{\prime }I\right]}^{-1}H^{T}\Lambda T \end{array}$$

where $\lambda^{\prime }=2N\lambda$, $\varphi \left(e_{i}\right)=\sum_{j=1}^{M}\frac{\alpha_{j}}{\sigma_{j}^{2}}G_{\sigma_{j}}{(e}_{i})$ and Λ is a diagonal matrix with diagonal elements $\Lambda_{ii}=\varphi \left(e_{i}\right)$, which provides the local similarity measurements between the predicted output and the actual outputs. When the training data contain large noise or many outliers, the corresponding diagonal elements are relatively low which induce the effects of such samples. Therefore, the algorithm can achieve high robustness against noises and outliers in the signals.

Since Equation (37) is a fixed-point equation because the diagonal matrix depends on the weight vector, the optimal solution should be solved by applying the evolved cooperation using Equation (37).

Therefore, combined with the kernel optimization in Section 3, the whole training process can be summarized in Algorithm 2, which is referred to as the ECC-ELM algorithm in this paper.

**Algorithm 2** ECC-ELM**Input:** the samples {x_i_,t_i_}, i = 1, 2, …, N **Output:** output weights **Parameters:** the number of hidden nodes N, the number of iterations L, the iterations T and termination tolerance ε **Initialization:** Randomly set the weights and bias of the hidden nodes and initialize the output weights β using Equation (5) 1: **for** t = 1, 2, …, T **do** 2:    Calculate the residual error: ei = t_i_ − ***h***_i_β, i = 1, 2, …, N 3:    Calculate the kernel parameters {σ,A} using Algorithm 1 4:    Calculate the diagonal matrix **Λ:**
$\Lambda_{ii}=\varphi \left(e_{i}\right)=\sum_{j=1}^{M}\frac{\alpha_{j}}{}G_{\sigma_{j}}{(e}_{i})$ 5:    Update the output weight using Equation (37) 6:    **Until** |J_k_(β) − J_k−1_(β)| < ε 7: **end for**

## 5. Analysis on Time Complexity and Space Complexity of ECC-ELM

In this section, the time complexity of the proposed method is analyzed and compared with the other algorithms. The main time complexity of the ECCELM comes from the cooperating evolution process and the training process of the model. The cooperative evolution contains the calculations of the influence coefficients and the particles updating with the time complexity of O(I_t_NK^2^), where I_t_ is the number of iterations, N is the number of particles and K is the number of disperse values of the outputs. To train the ELM, the procedures share the same time complexity as the RCC-ELM and MMCC-ELM, which is O(I_h_N_l_(5M+M^2^)), where I_h_ is the amount of iterations for training and N_l_ is the number of training data. Additionally, M is the number of hidden nodes. Therefore, the time complexity of ECC-ELM is O(I_h_N_l_(5M+M^2^+I_t_NK^2^)), which is slightly higher than those of the RCC-ELM and MMCC-ELM but it satisfies the requirements in most applications.

With respect to the spatial complexity, the ECC-ELM has the same complexity as the prediction models using the RCC-ELM, which is O(N+(N+2)M+N_l_^2^). Additionally, the space complexity consumed by evolving process is O(2N+K). Therefore, the space complexity of ECC-ELM is O(N+(N+2)M+N_l_^2^+2N+K), which has the same order as RCC-ELM and MMCC-ELM.

In summary, the time complexity and spatial complexity are practical for most applications.

## 6. Experiments

### 6.1. The Simulation of the Sinc Function with Sas noises

In this section, the simulation experiments using the Sinc function with random noises are presented. They compare between serval state-of-art algorithms with the proposed method, which are the R-ELM, the RCC-ELM, the MMCC-ELM and our method. The training and test samples were randomly assigned according to the Sinc function and random noises were added with respect to alpha-stable distribution. This is represented as follows:

(40) y=αSinc(x)+ρ

where α is the scale of the function which is set to 8.0 and Sinc(x) is the Sinc function. The Sinc function is represented as follows:

(41) $$\mathrm{Sinc}\left(x\right)=\{\begin{array}{c} \begin{array}{cc} \sin\left(x\right)/x & x\neq 0 \end{array}\\ \begin{array}{cc} 1 & x=0 \end{array} \end{array}$$

Moreover, ρ is the noise that satisfies the following characteristic function [69]:

(42) $$\rho =\{\begin{array}{c} \begin{array}{cc} \exp\left(-\delta^{\alpha }{\left|\theta \right|}^{\alpha }\left(1-j\beta sign\left(\theta \right)\tan\left(\frac{\pi \alpha }{2}\right)\right)\right)+j\mu \theta & \alpha \neq 1 \end{array}\\ \begin{array}{cc} \exp\left(-\delta^{1}{\left|\theta \right|}^{1}\left(1-j\beta \left(\pi /2\right)sign\left(\theta \right)\log\left(\frac{\pi \alpha }{2}\right)\right)\right) & \alpha =1 \end{array} \end{array}$$

The parameters α, β, γ and μ are real and characterize the distribution of the random variable X. Here, the alpha-stable probability distribution function is denoted as S(α,β,γ,μ). In these experiments, the four parameters were assigned to three different conditions to provide three types of noises. The assignment of the parameters in each sample is presented in Table 2.

Each sample contained 200 data, with half of the data being used for training and another half for testing. To get a proper estimation of the performances of each method, the experiments were operated with the best optimization of parameters. This is presented in Table 3.

Each experiment was conducted 30 times and the averages were taken. The comparison of the accuracies of these algorithms is presented in Table 4. Compared with other algorithms, the R-ELM and ECC-ELM achieve lower mean square errors due to the advantages of the correntropy. The performance of R-ELM is relatively poor due to the effect of noises. The performance of MMCC-ELM also improved by the correntropy. However, since the fixed dimension of the correntropy, the accuracy can be badly influenced by unnecessary assignments on the second order of the bandwidth. Furthermore, it is clear that the proposed algorithm achieves the lowest training MSE, which means that it is the most accurate method for simulation of the Sinc function.

**Figure 3:**
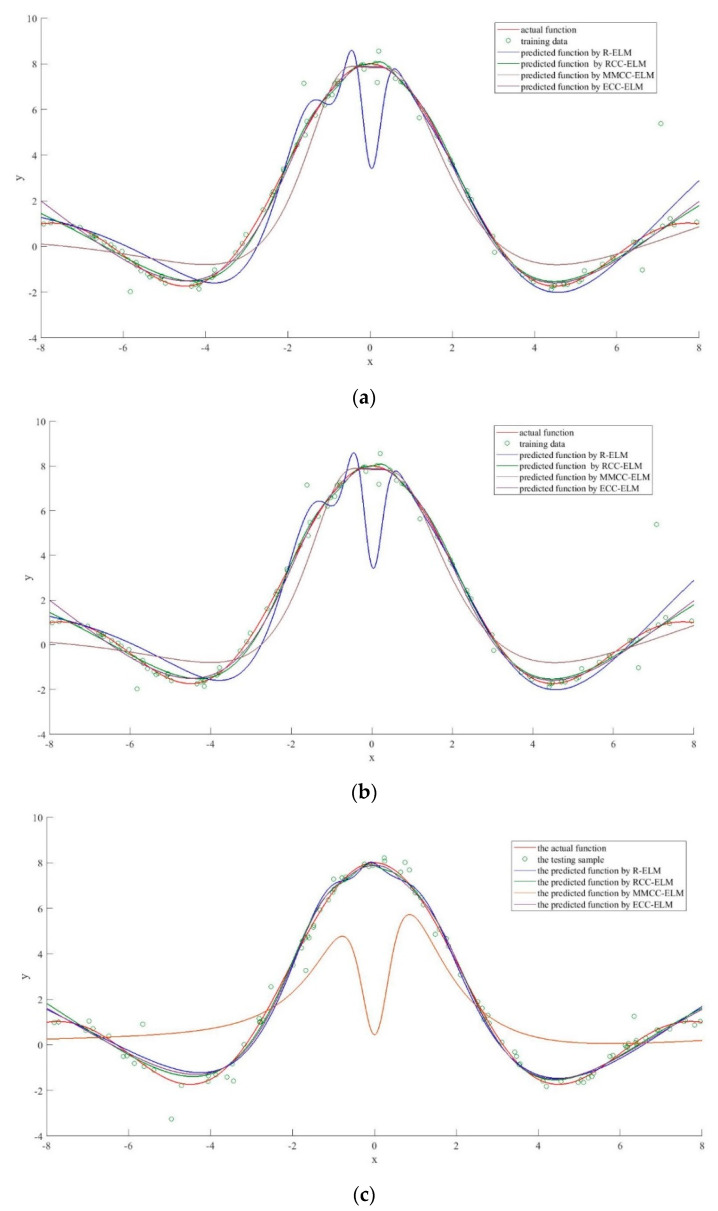
The performance comparison of each algorithm (**a**) comparison with sample 1; (**b**) comparison with sample 2 and (**c**) comparison with sample 3.

To further analyze the predictive abilities of these four algorithms, Figure 3 depicts the differences between the actual function and the predicted function for each algorithm. It is clear that all the algorithms achieve relatively good prediction on the Sinc function. However, the prediction results of the ELM have been badly influenced by the noises in all three samples. Additionally, the MMCC-ELM performance is poor on sample 2 and sample 3, which is probably due to the assignments with high dimension parameters. The RCC-ELM and ECC-ELM provide good predictions, which are almost identical to the actual functions in all three samples. The ECCELM has the closet predicted function with the Sinc function, which also proves that the method has high reliability against noise.

**Figure 4:**
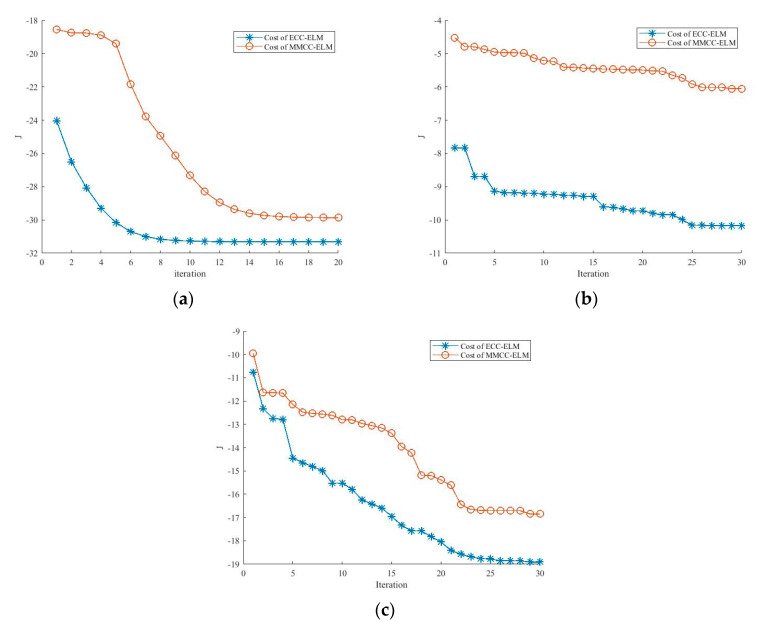
The comparison on the cost function values of the extreme learning machine by maximum mixture correntropy criterion (MMCC-ELM) and ECC-ELM (**a**) comparison with sample 1; (**b**) comparison with sample 2; (**c**) comparison with sample 3.

Furthermore, an experiment on sample 1 was conducted to compare the cost function for the output weights with the MMCC-ELM and ECC-ELM since they share similar cost functions. The results are shown in Figure 4, which show that the cost function of ECC-ELM is quite lower than the cost of MMCC-ELM. Additionally, the costs of the ECC-ELM become stable for less than 25 iterations for all three examples than MMCC-ELM. This shows the improvements on training the model with ECC-ELM taking the cooperating evolution technique. Since both algorithms finish the generation of the model when the cost function becomes stable, it can be concluded that the proposed model has faster convergence on training the prediction model.

**Figure 5:**
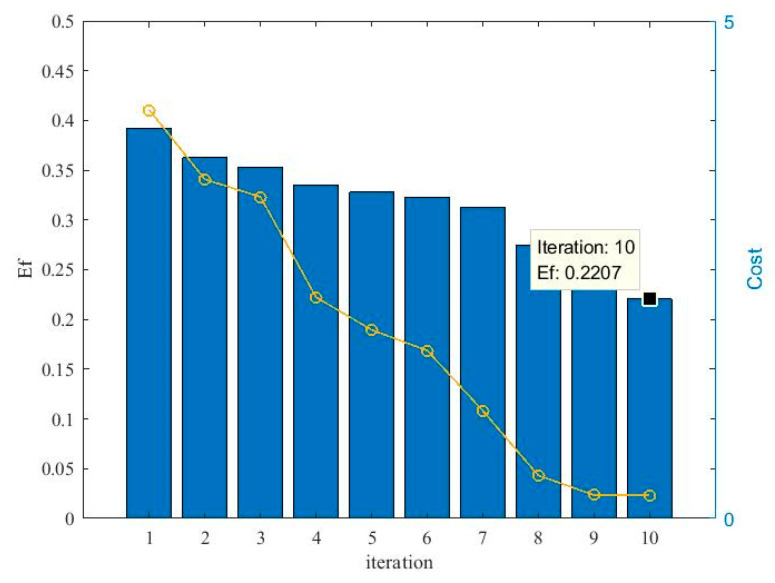
The dynamic changes of the evolution factor (Ef) and costs during the cooperative evolution.

**Figure 6:**
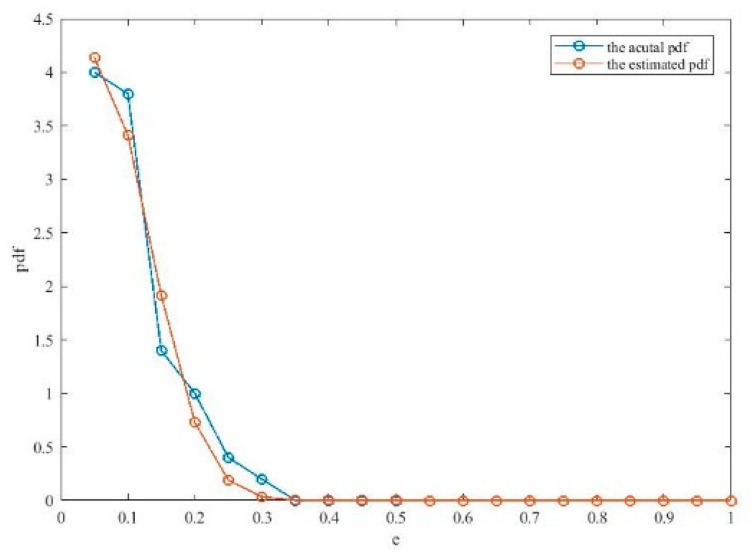
Comparison between the estimated pdf and actual pdf.

Figure 5 illustrates the effects of the evolutionary process on the optimization of the kernel bandwidth and influence coefficients. From Figure 5, it can be seen that the cost function for the kernel bandwidth quickly drops during the evolution process. Moreover, Ef continuously decreases during the process, which means that the particle swarm become stable and the best solution occurs. Figure 6 compares the actual pdf function and the estimated pdf function. It can be seen that the algorithm achieves a comparatively accurate estimation of the distribution of the errors.

### 6.2. The Performance Comparison on Benchmark datasets

To further assess the proposed algorithm, the performance of the ECC-ELM and other methods were compared using the data set from the UCI machine learning repository [70], awesome public dataset [71] and the United Nations development program [72], which are listed in Table 5. The assignments of the parameters are shown in Table 6, all of which refer to the best performance of each algorithm. Each experiment was conducted 30 times and the average performance was reported.

The performance is compared in Table 7, which shows that the proposed algorithm is able to achieve better prediction accuracies than other methods. Additionally, the performance of the proposed method is relatively stable compared with other correntropy-based extreme learning machines.

**Figure 7:**
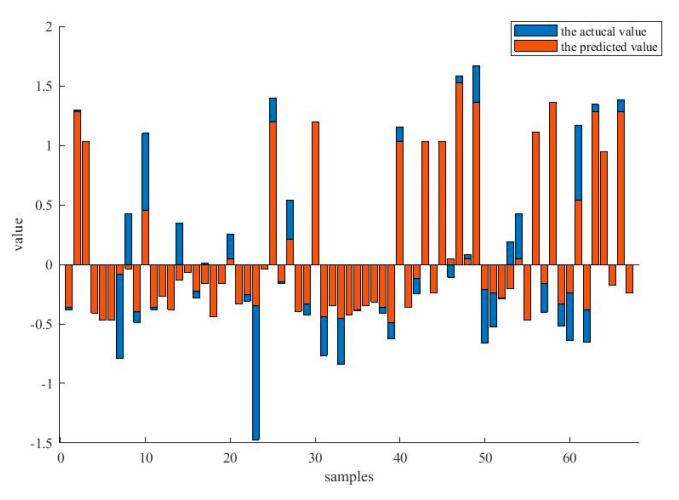
The comparison between the actual values and the predicted values under the data set, Servo.

Figure 7 compares the actual output value and the predicted value for the Servo data set. It is clear that the predicted values are basically identical to the actual output values, and it has not been influenced by the outliers in the data.

**Figure 8:**
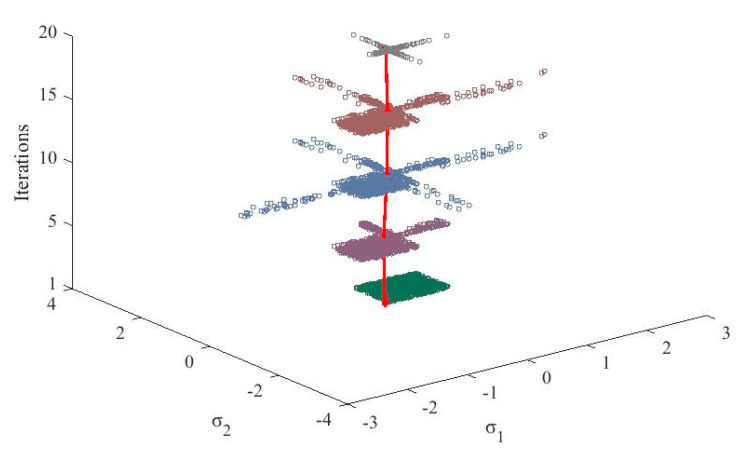
The evolutionary process of the particles.

To illustrate the evolutionary processes for optimizing the bandwidth, Figure 8 depicts the distributions of the particles and the evolution of the optimal solutions. It can be seen that the distribution of the particles dynamically changes based on the state of the PSO process. The optimal solution is adjusted and stabilizes during the process, which allows the optimal solution of the bandwidth assignments to generate a more accurate model.

### 6.3. The Performance Estimations for Forecasting the CTR of Optical Couplers

Finally, to estimate the performance of a real application, the proposed method has been used to predict the current transfer ratio for optical couplers. This is one type of transmission device for electric signals and optical signals with wide applications to the isolation transfer of signals, A/D transmission, D/A transmission, digital communications and high-pressure control. For optical couplers, the CTR is an essential factor for estimating the operating status of optical couplers. In this section, the proposed method was used to give the predictions of CTR for the optical couplers to predict the health condition of the devices.

For the experiments, the degenerating signals of four optical couplers were recorded and transformed into the samples historical CTR value as input vectors and the CTR value of the next time as the expected output. The training data was the samples that were generated from the optical couplers’ records over the first ten years and the testing data were the samples that were generated from the last ten years.

**Figure 9:**
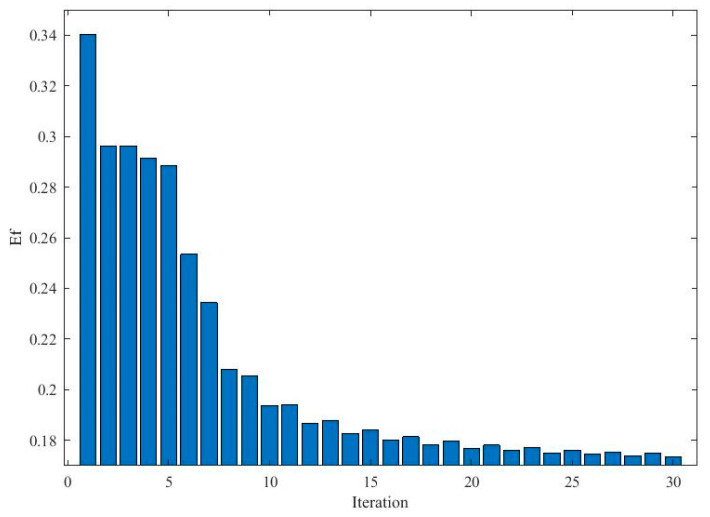
Dynamic changes on Ef and costs during the cooperative evolution.

Figure 9 depicts the evolutionary process of the PSO procedure. It shows that the Ef value quickly decreases during the evolutionary process and stabilizes within 17 iterations, resulting in the optimal solution that is provided by the swarm.

**Figure 10:**
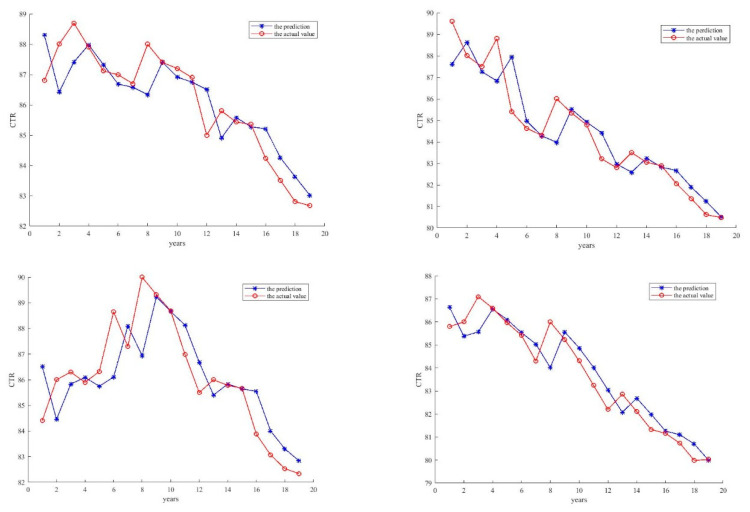
The comparison with the predicted current transfer ratio (CTR) and actual CTR.

Finally, the predicted results of the four optical couplers are shown in Figure 10. It is clear that the generated ELM network accurately predicts the CTR value of each optical coupler and is robust with the noises of the signals. Therefore, the proposed method is able to achieve good performance for the optical couplers.

Table 8 presents the numerical results of the CTR prediction, which compares the actual CTR and the predicted CTR. It is clear that the proposed method can very accurately provide the prediction on the state of Optical Couplers (OCs). Additionally, the time consumption is presented in Table 8 which shows that the proposed method is able to obtain high accuracy on the prediction of the future CTR of the OC and the predicting time is quite low within 5 ms. Therefore, the proposed method can achieve high performance on real applications.

## 7. Conclusions

To improve the robustness of the forecasting model, the paper provides a novel correntropy-based ELM called the ECC-ELM. It uses a multi-dimension correntropy criterion and the evolved cooperation method to adaptively generate the parameters for kernels. In the proposed algorithm, SDPSO is integrated by minimizing the MIE to determine the proper bandwidths and their corresponding influence coefficients to estimate the probability distributions of the residual error of the model. A novel training process was developed based on the properties of the multi-dimension correntropy and it was able to build the convex cost function to calculate the output weights for the ELM. The experiments on the simulated data and real-world application were conducted to estimate the accuracy of the probability distribution of the signal and robustness on predicting the samples. The simulation results with the Sinc function proved that the proposed method can generate the multi-kernel correntropy with high accuracy on describing the probability distribution of the signals and fast converge on the evolution process. This leads to high robustness of the proposed method compared with the other methods. The performance comparisons on the benchmark datasets show that the proposed method can achieve higher accuracy and more stability than the other methods. Finally, the CTR prediction experiments show the proposed method can achieve high accuracy within acceptable time consumption on real world applications. Although the proposed algorithm has predictive advantages, there are still several limitations on the study. One limitation is the proposed method is only applicable for an ELM with one hidden layer, which requires extensions on multi-layer networks. The other limitation is that the proposed method only provides an offline training model. Therefore, how to update the online prediction model becomes another interesting topic for future research. The codes and data of the research are available at https://github.com/mwj1997/ECC-ELM.

## Figures and Tables

**Table 1:** The strategies for selecting the parameters.

State	Range of Ef	c_1_	c_2_	pσ	gσ	$\tau_{1}$	$\tau_{2}$
Convergence	0≤Ef<0.25	2	2	pσ(k)	gσ(k)	0	0
Exploitation	0.25≤Ef<0.5	2.1	1.9	$p\sigma \left({k-\tau }_{1}\left(k\right)\right)$	gσ(k)	$\left[k\cdot rand_{1}\right]$	0
Exploration	0.5≤Ef<0.75	2.2	1.8	pσ(k)	$g\sigma \left({k-\tau }_{2}\left(k\right)\right)$	0	$\left[k\cdot rand_{2}\right]$
Jumping out	Ef > 0.75	1.8	2.2	$p\sigma \left({k-\tau }_{1}\left(k\right)\right)$	$g\sigma \left({k-\tau }_{2}\left(k\right)\right)$	$\left[k\cdot rand_{1}\right]$	$\left[k\cdot rand_{2}\right]$

**Table 2:** The assignments of the parameters in each sample.

Sample #	α	β	γ	μ
Sample 1	1	0	0.001	0
Sample 2	0.7	0	0.0001	0
Sample 3	1.2	0	0.001	0

**Table 3:** The assignment of the parameters for each algorithms.

Algorithm	Parameter	Sample 1	Sample 2	Sample 3
R-ELM	N	100	100	100
	λ	0.00001	0.0001	0.0001
RCC-ELM	N	100	100	100
	λ	0.00001	0.00001	0.00001
	I_hq_	30	30	30
	ε	0.0001	0.0001	0.0001
	σ	1	1.2	1.2
MMCC-ELM	N	100	100	100
	λ	0.00001	0.00001	0.00001
	I_hq_	30	30	30
	ε	0.0001	0.0001	0.0001
	Σ_1_	2	2.2	4.3
	Σ_2_	0.8	0.8	8.5
	α	0.8	0.8	0.9
ECC-ELM	N	100	100	100
	λ	0.00001	0.00001	0.00001
	I_hq_	30	30	30
	ε	0.0001	0.0001	0.0001

**Table 4:** The comparison of the accuracies of the four algorithms.

Samples	ELM		RCC-ELM		MMCC-ELM		ECCC-ELM	
	Training MSE	Testing MSE	Training MSE	Testing MSE	Training MSE	Testing MSE	Training MSE	Testing MSE
Sample 1	0.336	0.6601	0.1339	0.3505	0.7225	1.1085	0.1415	0.3595
Sample 2	0.0828	0.11	0.0507	0.0892	1.363	2.189	0.0257	0.0576
Sample 3	0.2219	0.2572	0.2076	0.2339	0.868	0.7583	0.2046	0.2237

**Table 5:** The information on the data sets.

Data Set	Features	Observations	
		Training Numbers	Testing Numbers
Servo	5	83	83
Slump	10	52	51
Concrete	9	515	515
Housing	14	253	253
Yacht	6	154	154
Airfoil	5	751	751
Soil moisture	124	340	340
HDI	12	93	93
HIV	10	65	65

**Table 6:** Parameter settings of each algorithm.

Algorithm	Parameter	Servo	Slump	Concrete	Housing	Yacht	Airfoil	Soil Moisture	HDI	HIV
R-ELM	N	90	190	185	180	185	200	200	100	100
	λ	0.00010000	0.00050000	0.00020000	0.00020000	0.00002000	0.00002000	0.00002000	0.00001000	0.00001000
RCC-ELM	N	120	100	200	200	200	180	180	150	120
	λ	0.00001000	0.00010000	0.00000100	0.00010000	0.00000001	0.00000001	0.00000001	0.00000001	0.00000001
	I_hq_	30	30	30	30	30	30	30	30	30
	ε	0.00010000	0.00010000	0.00010000	0.00010000	0.00010000	0.00010000	0.00010000	0.00010000	0.00010000
	σ	0.00100000	0.00001000	0.00005000	0.01000000	0.00000100	0.00000100	0.00000100	0.00000100	0.00000130
MMCC-ELM	N	90	165	200	200	195	150	150	150	150
	λ	0.00100000	0.00001000	0.00005000	0.01000000	0.00000100	0.00000100	0.00000100	0.00000100	0.00000100
	I_hq_	30	30	30	30	30	30	30	30	30
	ε	0.00010000	0.00010000	0.00010000	0.00010000	0.00010000	0.00010000	0.00010000	0.00010000	0.00010000
	Σ_1_	0.2	0.5	0.5	0.5	0.5	0.2	1.0	1.2	0.7
	Σ_2_	2.8	1.6	2.6	2	2	2.7	0.7	0.8	0.3
	α	0.8	0.3	0.5	0.8	0.8	0.5	0.6	0.7	0.6
ECC-ELM	N	90	180	180	180	180	200	200	200	200
	λ	0.00100000	0.00001000	0.00005000	0.01000000	0.00000100	0.00000100	0.00000100	0.00000100	0.00000100
	I_hq_	30	30	30	30	30	30	30	30	30
	ε	0.00010000	0.00010000	0.00010000	0.00010000	0.00010000	0.00010000	0.00010000	0.00010000	0.00010000

**Table 7:** The performance comparison.

Data Set	R-ELM		RCC-ELM		MMCC-ELM		ECCC-ELM	
	Training RMSE	Testing RMSE	Training RMSE	Testing RMSE	Training RMSE	Testing RMSE	Training RMSE	Testing RMSE
Servo	0.0590 ± 0.009	0.1039 ± 0.0164	0.0740 ± 0.0106	0.1031 ± 0.0148	0.0839 ± 0.0174	0.0989 ± 0.0187	0.1047 ± 0.0181	0.8742 ± 0.0131
Slump	0.0081 ± 0.0011	0.0461 ± 0.0095	0.0000 ± 0.0000	0.0422 ± 0.0094	0.0001 ± 0.0000	0.0408 ± 0.0101	0.0001 ± 0.0001	0.354 ± 0.1890
Concrete	0.0738 ± 0.0021	0.0917 ± 0.0045	0.0561 ± 0.0018	0.0872 ± 0.0066	0.0560 ± 0.0021	0.0867 ± 0.0064	0.0561 ± 0.0018	0.0852 ± 0.0053
Housing	0.0439 ± 0.0043	0.0896 ± 0.0124	0.0495 ± 0.0045	0.0830 ± 0.0110	0.0554 ± 0.0045	0.0821 ± 0.0101	0.0352 ± 0.0013	0.0791 ± 0.0110
Yacht	0.0366 ± 0.0093	0.0529 ± 0.0090	0.0125 ± 0.0008	0.0349 ± 0.0113	0.0125 ± 0.0008	0.0328 ± 0.0074	0.0172 ± 0.0027	0.0268 ± 0.0031
Airfoil	0.0974 ± 0.0074	0.1031 ± 0.0077	0.0736 ± 0.0022	0.0906 ± 0.0054	0.0736 ± 0.0025	0.0898 ± 0.0051	0.0736 ± 0.0023	0.0889 ± 0.0046
Soil moisture	0.0032 ± 0.0011	0.0095 ± 0.0013	0.0007 ± 0.0001	0.0015 ± 0.0003	0.0006 ± 0.0000	0.0012 ± 0.0002	0.0006 ± 0.0000	0.0009 ± 0.0001
HDI	0.0004 ± 0.0001	0.0006 ± 0.0002	0.0001 ± 0.0000	0.0003 ± 0.0001	0.0001 ± 0.0000	0.0003 ± 0.0001	0.0001 ± 0.0000	0.0003 ± 0.0001
HIV	0.0376 ± 0.0220	0.0599 ± 0.0130	0.0050 ± 0.0017	0.0079 ± 0.0009	0.0047 ± 0.0006	0.0065 ± 0.0004	0.0059 ± 0.0007	0.0059 ± 0.0006

**Table 8:** The performance of the predicted model that is generated using the ECCELM.

Time (year)	Actual CTR	Predicted CTR	Normalized Error	Predicting Time (ms)
1	87.90	88.03	0.0037	2.98
2	87.70	88.01	0.0068	4.02
3	87.40	87.94	0.0274	3.92
4	85.50	87.15	0.0095	4.98
5	86.30	87.02	0.0122	2.26
6	85.93	86.61	0.0084	3.22
7	85.86	85.40	0.0188	5.74
8	84.73	85.30	0.0145	4.48
9	84.01	84.33	0.0115	5.85
10	83.31	83.38	0.0023	4.87
